# Prematurely delivering mothers show reductions of lachnospiraceae in their gut microbiomes

**DOI:** 10.1186/s12866-023-02892-z

**Published:** 2023-06-15

**Authors:** Ru Yang, Xiaoyu Li, Zhiye Ying, Zicheng Zhao, Yinan Wang, Qingyu Wang, Bairong Shen, Wentao Peng

**Affiliations:** 1 Department of Neonatology Nursing, West China Second University Hospital, Sichuan University/West China School of Nursing, Sichuan University, Chengdu, China; 2 Key Laboratory of Birth Defects and Related Diseases of Women and Children (Sichuan University), Ministry of Education, Chengdu, China; 3 Institutes for Systems Genetics, Frontiers Science Center for Disease-related Molecular Network, West China Hospital, Sichuan University, Sichuan, China; 4 West China Biomedical Big Data Center, West China Hospital, Sichuan University, Sichuan, China; 5 Medical Big Data Center, Sichuan University, Chengdu, Sichuan China; 6 Shenzhen Byoryn Technology, Shenzhen, Guangdong P.R. China; 7 Peking University Shenzhen Hospital, 1120 Lianhua Road, Shenzhen, China; 8 School of Business Administration, Northeast University, Shenyang, China

**Keywords:** Preterm birth, Gastrointestinal microbiota, Shotgun metagenomics sequencing, Lachnospiraceae, Short-chain fatty acids, Interleukin- 1

## Abstract

**Background:**

Preterm birth is the leading cause of perinatal morbidity and mortality. Despite evidence shows that imbalances in the maternal microbiome associates to the risk of preterm birth, the mechanisms underlying the association between a perturbed microbiota and preterm birth remain poorly understood.

**Method:**

Applying shotgun metagenomic analysis on 80 gut microbiotas of 43 mothers, we analyzed the taxonomic composition and metabolic function in gut microbial communities between preterm and term mothers.

**Results:**

Gut microbiome of mothers delivering prematurely showed decreased alpha diversity and underwent significant reorganization, especially during pregnancy. SFCA-producing microbiomes, particularly species of *Lachnospiraceae, Ruminococcaceae*, and *Eubacteriaceae*, were significantly depleted in preterm mothers. *Lachnospiraceae* and its species were the main bacteria contributing to species’ differences and metabolic pathways.

**Conclusion:**

Gut microbiome of mothers delivering prematurely has altered and demonstrates the reduction of *Lachnospiraceae*.

**Supplementary Information:**

The online version contains supplementary material available at 10.1186/s12866-023-02892-z.

## Introduction

Preterm birth (PTB) is defined as birth before 37 weeks. Fifteen million infants were born preterm worldwide, which puts these children at risk of morbidity and mortality or long-term health problems [1, 2]. Complications of PTB were the leading cause of death among children under five years [3]. The potential lifelong impact lasts into adolescence and even adulthood, affecting their motor, learning abilities, mental health, and social functioning [4, 5].

The etiology of PTB is still unclear. There is consensus that PTB is a syndrome involving multiple biological pathways [6]. The pro-inflammatory cytokines, especially interleukin-1 (IL-1) are known to contribute to inflammation-induced PTB critically [7]. While Maternal-fetal genetics and immunity have provided substantial insights into the molecular mechanism of PTB [8], accumulating evidence indicates that the host gut microbiome regulates maternal and fetal immune interaction and directly affects the birth outcome [9]. The dysbiosis of the maternal microbiome affects metabolism and inflammatory processes through microbiome-generated metabolites, which may induce the PTB [10]. The association between vaginal [11], oral microbiome [12] with PTB has been widely reported. An intrauterine infection ascending from the vagina is widely characterized as an important contributor to the onset of preterm labour. The vaginal microbiome of women who delivered term is dominated by *Lactobacillus* during pregnancy [13]. In contrast, women who delivered preterm exhibited significantly lower vaginal levels of *Lactobacillus*[14]. Depletion of *Lactobacillus* species and a more diverse vaginal microbiome leads to increased mannose binding lectin, IgM, IgG, C3b, C5, IL-8, IL-6, IL-1β and increased risk of PTB [15]. Haematogenous dissemination of microbes from oral cavity has also been proposed as another potential route of infection leading to PTB [12]. However, despite a number of observational studies show an increased risk of PTB in women with periodontitis diseases, the evidence behind it is still weak [16, 17].

We know little about the potential interaction effects of the maternal gut microbiome on PTB. Only a few studies identified the distinct gut microbiome in preterm mothers. Shiozaki et al. [18] showed a reduction of several species from *Clostridium* and *Bacteroides* were observed in the gut of mothers delivering prematurely. Cecilie Dahl et al. [19] found that mothers of preterm deliveries show reduced diversity and lower relative abundance of *Bifidobacterium* and *Streptococcus*. In a recent study, Chunhua Yin et al. [20] reported that gut microbiome of patients who deliver preterm contains higher common oral bacteria. While these studies were provided by 16 S rRNA. Shotgun metagenomics improves the ability to discriminate microbiome at a species-level, or even strain-level, and allows accurate functional annotation of the gene sequences [21]. Compared to previous articles, our study performed shotgun metagenomic sequencing to identify a greater number of genera. A total of 765 genera were detected, and in addition, 575 distinct species were identified at the species level. Thus, high-resolution metagenomic studies are required to examine the impact of disrupted gastrointestinal bacteria in the maternal gut on the PTB.

Here, we perform a metagenomic analysis to identify the taxonomic composition and metabolic function in gut microbial communities between preterm and term mothers. We also explore how the maternal gut microbiome may contribute to PTB progression.

## Method

### Subject recruitment and sample collection

This study was approved by the Ethical Committee of the West China Second Hospital of Sichuan University (2,021,112). Informed written consent was obtained from all the participants. Forty-three mothers were recruited at the West China Second Hospital of Sichuan University from December 2020 to December 2021. We excluded mothers with metabolic diseases, such as obesity, diabetes, hypertension, and hyperuricemia, and mothers with immune diseases, such as rheumatoid arthritis, lupus erythematosus, Hashimoto’s thyroiditis, and ulcerative colitis. We also excluded mothers who do not want to participate in the study or withdraw from the study.

Before sample collection, we explained the collection precautions to the mothers in detail. We sampled the mother’s fecal samples during the last week of pregnancy and one month after delivery. Samples collected in the hospital were immediately sent to the laboratory and frozen at -20 ℃, then transferred to store at − 80 ℃ within 24 h. The samples collected at home were shipped to the laboratory in West China Second Hospital of Sichuan University within 2–3 days, then the samples were stored at -80℃. The instruction of stool collection tubes (Shbio scRNA-seq Kit, shanghai, china) showed the samples could store at room temperature for 14 days (https://www.shbio.com/products/3033).

### Fecal genomic DNA extraction and sequencing

The microbial community DNA was extracted using QIAamp PowerFeacal Pro DNAKit (QIAGEN, 51804, USA) following the manufacturer’s instructions. DNA concentration was measured using a NanoDrop spectrophotometer (Thermo Fisher Scientific, USA) and Qubit Fluorometer (Invitrogen, USA). All extracted DNA samples were stored at − 80°C until shotgun metagenomic sequencing. We did negative controls in the same way as the actual samples. DNA was randomly fragmented by Covaris. The fragmented DNA was selected by Magnetic beads to an average size of 200–400 bp. The selected fragments were through end-repair, 3’ adenylated, adapters-ligation, and PCR Amplifying, and the products were purified by the Magnetic beads. The double-stranded PCR products were heat denatured and circularized by the splint oligo sequence. The single-strand circle DNA (ssCir DNA) was formatted as the final library and qualified by QC. Paired-end sequencing was performed on the DNBSEQ-T7 platform (BGI-Shenzhen, China) with an insert size of 350 bp and paired-end (PE) reads of 150 bp, targeting ~ 10 Gb of sequence per sample.

### Quality control

We performed quality control of the sequencing data using KneadData (http://huttenhower.sph.harvard.edu/kneaddata) with default parameters. Adapters were removed and reads with low quality bases were trimmed (windows: 4-mer, Phred quality < 20) and truncated (< 50% of pre-trimmed length) using Trimmomatic (version = 0.39). The filtered paired-end reads were mapped to the human genome (hg19, GCA_000001405.1) using bowtie2 (version = 2.3.1) with default parameters (--very-sensitive) to eliminate potential human contamination.

### Taxonomy annotation and functional annotation

Taxonomic and functional profiling of metagenomic sequencing data was performed with the wmgx bioBakery workflow built with AnADAMA2 [22] (http://huttenhower.sph.harvard.edu/biobakery). Taxonomic profiling was conducted using MetaPhlAn3 (version = 3.0.14) with the default parameters. Functional profiling was performed by HUMAnN3 (version = 3.0.1) with the default parameters. The CHOCOPhlAn (release 2019.01) database was used for taxonomy and functional annotation, whereas the UniRef90 database (release 2021.03) was used for gene family abundance determination.

### Bioinformatics analysis

We used Wilcoxon rank-sum test to compare demographics characteristics between groups. The ‘amplicon’ R package (Version: 3.5.3) was used to visualize the rarefaction curve. We used the Gutmeta website (https://gutmeta.deepomics.org/) to visualize the richness and evenness of the gut microbiome composition with the Shannon index. We used principal coordinates analysis (PCoA) of binary Bray-Curtis distance to evaluate the gut microbiota from different groups, which was calculated by USEARCH. We conducted PERMANOVA with Bray-Curtis distance using the ‘adonis’ function from the ‘vegan’ R package (Version: 3.5.3). The ‘ggplot2’ package (Version: 3.5.3) was used to visualize the relative abundance of phylum, genus, and species levels. The potential biomarkers were assessed with the LEfSe method. The effect size of differentially abundant genera was estimated by linear discriminant analysis (LDA)[23]. The LDA score > 2.0 and p-value < 0.05 were considered as statistically significant. Statistical Analysis of Metagenomic Profiles (STAMP) software was used to conduct significantly different bacteria between groups with Wilcoxon [24]. HUManN2-associated module was used to identify differentially functional pathways. All the p values were adjusted by False Discovery Rate (FDR) and q-values < 0.05 were considered as statistically significant.

## Result

### Study population

**Fig. 1:**
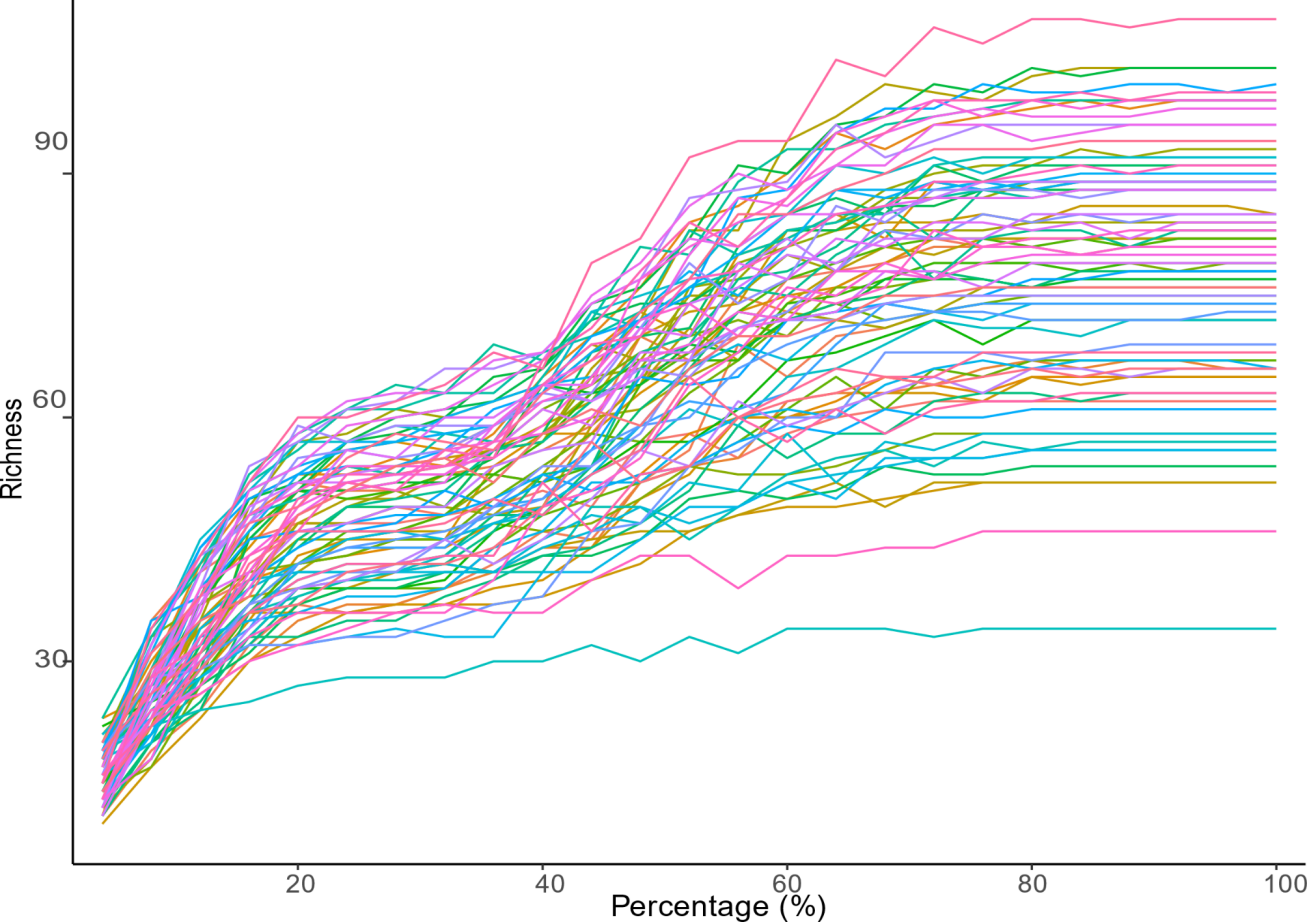
Rarefaction Curve

To interrogate the composition and function of the gut microbiome between preterm mothers (PMs) and term mothers (TMs), we enrolled 43 mothers with 33 TMs and 10 PMs. During the last week of pregnancy, we collected 28 TMs and 9 PMs, and one month after delivery, we collected 33 TMs and 10 PMs. The demographic information of the participants is presented in Table 1. No significant differences were observed in maternal age, pregnancy weight, and prenatal BMI between the TMs and PMs groups (Table 1; *P* > 0.05). There was a significant difference in gestational age and birth weight between the two groups, with the PMs group showing a lower total score compared to the TMs group (Table 1; *P* < 0.001). The Rarefaction Curve showed that the curve tends to be flat as the sequencing depth increased, which indicated the sequencing depth is sufficient (Fig. 1).

**Table 1:** Clinical characteristics of TMs and PMs

Variables	TMs	PMs	*p-value*
Maternal age(years)	30.88 ± 3.328	31.40 ± 2.591	0.654
Pregnancy Weight (kg)	63.838 ± 6.490	62.790 ± 7.726	0.669
Prenatal BMI(kg/m^2^)	25.15 ± 2.476	24.56 ± 2.044	0.497
Gestational age (days)	268.53 ± 7.609	251.2 ± 7.239	<0.001
Birth weight (g)	3027.21 ± 332.172	2456 ± 436.949	<0.001

The statistical significance was evaluated by *t* test for independent samples for continuous variables.

### Distinct gut microbial communities in PM

**Fig. 2:**
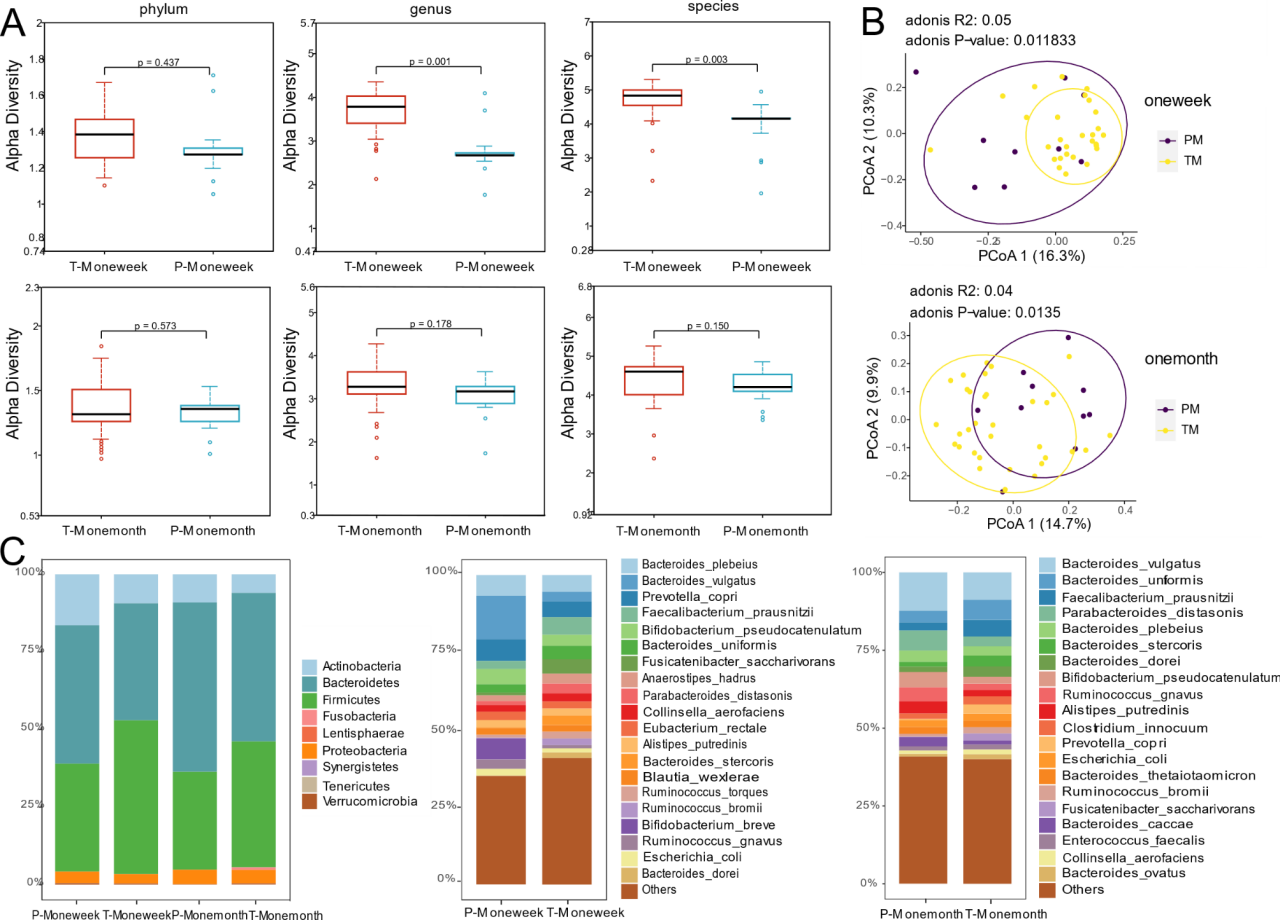
Distinct Gut Microbial Communities in Mothers Delivering Prematurely. **(A)** Comparison of alpha diversity between the PMs and TMs groups at phylum, genus, and species level. **(B)** Principal coordinates analysis (PCOA) analysis between the PMs and TMs groups. **(C)** Relative abundances of gut microbiomes for PMs and TMs at phyla and species level

We first analyzed the alpha diversity of communities at different periods. During the last week of pregnancy, there was no difference in alpha diversity at the phylum level (p > 0.05). However, the alpha diversity was decreased in the preterm group at the genus and species levels (p < 0.01). After delivery, no discernable differences between the two groups, either at phylum, gene level, or species level, were identifiable (p > 0.05; Fig. 2A). These results indicate that a high richness and evenness in the TMs group and the gut of PMs altered in the third trimester of pregnancy but soon returned to the normal level after delivery. Beta-diversity was calculated using bray-Curtis distance metrics to measure the extent of similarity in fecal microbial communities (Fig. 2B). Principal coordinate analysis (PCoA) revealed separation of the two groups before (PERMANOVA, P = 0.011) and after delivery (PERMANOVA, P = 0.013). The analysis indicated that the microbiome community of the PMs group was heterogeneous and significantly different from that of the TMs group.

We next sought to understand how microbiome composition and developmental trajectory were altered. Mothers had a high fraction of *Firmicutes* and *Bacteroidetes*, as expected for a healthy China pregnancy gut. Compared with TMs, PMs showed an increase in *Bacteroidetes* and a decrease in *Firmicutes*, especially during pregnancy. We confirmed this observation on the species-level profiles. During pregnancy, TMs were dominated by species from the *Bacteroides* (28.3%) and *Clostridia* (23.3%). In contrast, there was a significant decrease of *Clostridia* (14.5%) in PMs, including *Faecalibacterium prausnitzii, Fusicatenibacter saccharivorans*, *Anaerostipes hadrus*, *Ruminococcus torques* and *Ruminococcus bromii*, and most of which were belonged to *Lachnospiraceae* (11.7%). The differences in microbial species between the two groups were reduced after delivery (Fig. 2C).

### Significant reductions of SFCAs-producing microbiomes in mothers delivering prematurely

**Fig. 3:**
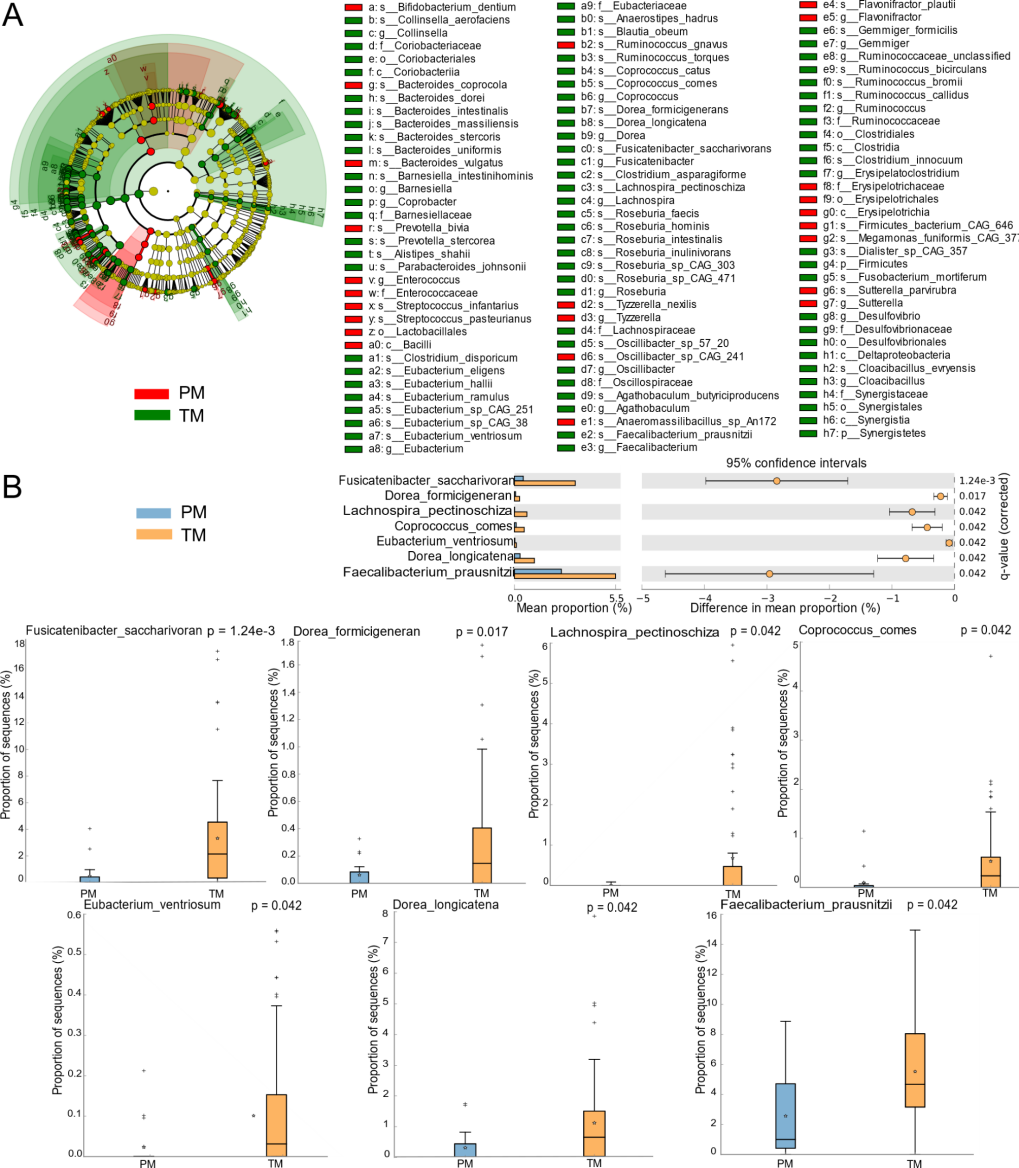
Significant reductions of SFCAs-Producing microbiomes in PMs. **(A)** LEfSe analysis of PMs and TMs groups. **(B)** Statistically significant different species in PMs and TMs groups

Linear discriminate analysis effect size (LEfSe) was also performed to corroborate representative taxa further. We performed LefSe analysis and identified 116 taxa showing significant differences (P < 0.05, LDA > 2), 92 taxa in TMs, and 24 in PMs (Fig. 3A, Supplementary Figure S1). The *Lachnospiraceae, Ruminococcaceae, Eubacteriaceae*, *Prevotellaceae* and members of these bacterium such as *Faecalibacterium prausnitzii, Bacteroides. uniformis, Bacteroides. stercoris, Fusicatenibacter. saccharivorans, Bacteroides dorei, Anaerostipes hadrus, Ruminococcus bromii, Eubacterium eligens, Ruminococcus torques*, and *Blautia obeum* were signature taxa for TMs. While PMs were characterized by the *Bacteroidaceae, Erysipelotrichaceae*, and *Pasteurellaceae*, such as *Bacteroides thetaiotaomicron, Ruminococcus gnavus, Bacteroides vulgatus, Megamonas funiformis, Sutterella parvirubra*, and *Streptococcus infantarius*. The results demonstrated that the communities of TMs were dominated by species from *Lachnospiraceae, Ruminococcaceae*, and *Eubacteriaceae*, which are efficient degraders of dietary fibers and producers of short-chain fatty acids (SCFAs). These SFCAs-producing species were deficient in the gut of PMs.

Given the differences of composition in the maternal gut metagenomes, we sought to validate the diversity of species between PMs and TMs in the gut microbiome (Fig. 3B). A total of seven different species were identified, including *Fusicatenibacter saccharivorans, Dorea formicigenerans, Lachnospira pectinoschiza, Coprococcus comes, Eubacterium ventriosum, Dorea longicatena*, and *Faecalibacterium prausnitzii* (p < 0.05). All of them belong to the *Clostridia* order. The *Eubacterium ventriosum* belongs to *Eubacteriaceae*, *Faecalibacterium prausnitzii* belongs to *Ruminococcaceae*, and the rest (71.4%, 5/7) belong to *Lachnospiraceae*.

### Differential microbial functions in the gut microbiome between PMs and TMs

**Fig. 4:**
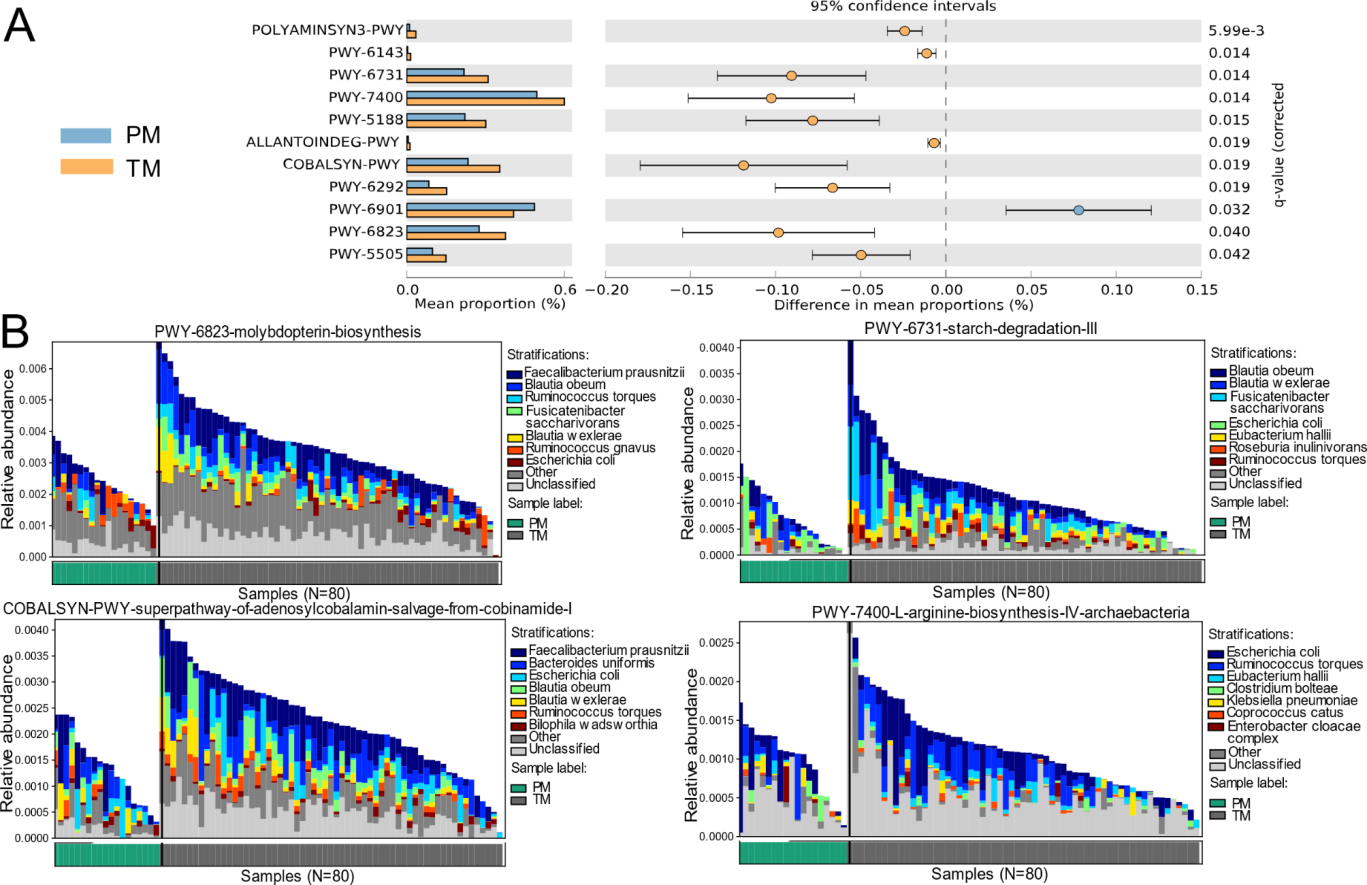
Differential microbial functions in the gut microbiome between PMs and TMs. **(A)** Statistically significant differences in predicated metabolic pathways between PMs and TMs groups. **(B)** Visualization of different species involved in differential microbial functions

To assess whether the apparent taxonomic differences between the gut microbiomes of PMs and TMs are reflected at the level of functional potential, we compare the functional profiles of PMs to those of healthy mothers. Several pathways were over-represented in TMs (p < 0.05), including amino acid, adenosylcobalamin, molybdenum pterin, and starch metabolism pathways (Fig. 4A). We found that some different species were involved the inmolybdopterin biosynthesis pathway, the starch degradation III pathway, the superpathway of adenosylcobalamin salvage from cobinamide pathway, and the L-arginine biosynthesis IV pathway among these metabolic pathways, so the four metabolic pathways were further visualized (Fig. 4B).

In the molybdopterin biosynthesis pathway, *Faecalibacterium prausnitzii, Blautia obeum, Ruminococcus torques, Fusicatenibacter saccharivorans, Blautia wexlerae, Ruminococcus gnavus, Escherichia coli* mainly contributed to this metabolism. Most of the bacterium belong to *Lachnospiraceae*, except the *Faecalibacterium prausnitzii*, which belongs to *Ruminococcaceae*. In the starch degradation III pathway, *Blautia obeum, Blautia wexlerae, Fusicatenibacter saccharivorans, Escherichia coli, Eubacterium hallii, Roseburia inulinivorans, Ruminococcus torques* mainly contributed to the pathway. Similarly, except *Eubacterium hallii* belongs to *Eubacteriaceae*, other species belong to *Lachnospiraceae*. In the superpathway of adenosylcobalamin salvage from cobinamide pathway, *Faecalibacterium prausnitzii, Bacteroides uniformis, Escherichia coli, Blautia obeum, Blautia wexlerae, Ruminococcus torques, Bilophila wadsworthia* contributed to the pathway, of which *Blautia obeum, Blautia wexlerae, Ruminococcus torques* belong to *Lachnospiraceae*. In the L-arginine biosynthesis IV pathway, *Escherichia coli*, *Ruminococcus torques*, *Eubacterium hallii*, *Clostridium bolteae*, *Klebsiella pneumoniae*, *Coprococcus catus*, *Enterobacter cloacae complex* contributed to the pathway. *Ruminococcus torques*, *Coprococcus catus* belong to *Lachnospiraceae*. These results indicate that *Lachnospiraceae* and its species were the main bacteria that contributed to the differential species and metabolic pathways in the gut of preterm mothers.

## Discussion

We collected fecal samples from preterm and term mothers, coupled with deep metagenomic sequencing, which allowed us to characterize these communities’ microbial composition and functions. We found that the gut microbiome of mothers delivering prematurely showed reduced alpha diversity and underwent significant reorganization, especially during pregnancy. Mothers delivering prematurely lacked species from *Lachnospiraceae, Ruminococcaceae, and Eubacteriaceae*, *Lachnospiraceae*, and its species were the main bacterium that contributes to the differential species and metabolic pathways in the gut of preterm mothers.

This is the first study comparing gut microbial communities between preterm and term mothers performing with shotgun metagenomics sequencing. A few studies have investigated the gut microbiome in preterm mothers performing 16 S rRNA amplicon sequencing. Shiozaki et al. [18] observed that the level of *Clostridium subcluster XVIII, Clostridium cluster IV*, and *Clostridium subcluster XIVa* were significantly lower in the mothers of preterm birth compared to non-preterm labor (term) group by using Terminal Restriction Fragment Length Polymorphism. A similar conclusion was found in another study. Cecilie Dahl et al. [19] found that mothers of preterm deliveries had a significantly lower abundance of Clostridium, including *Ruminococcaceae*, and *Mogibacteriaceae*, which agrees with our findings of a lower abundance of several OTUs from the *Clostridiale*s order. Chunhua Yin et al. [20] found that patients with preterm birth were enriched with opportunistic pathogens, particularly *Porphyromonas, Streptococcus, Fusobacterium*, and *Veillonella* 16 S rRNA amplicon sequencing. We did not find enriched opportunistic pathogens in preterm mothers. The reason may be the different sampling times between studies. Chunhua Yin collected fecal samples in the middle of pregnancy. In contrast, we sampled the fecal of mothers in the last week before delivery, and it is well known that the composition of the gut microbiome will change in different periods of pregnancy.

We found that the gut of mothers delivering prematurely was lack of *Lachnospiraceae, Ruminococcaceae, and Eubacteriaceae*. These bacteria are abundant anaerobic bacteria of gut microbes, producing SCFA from dietary fiber degradation [25]. SCAFs are the primary energy source for colonocytes, which play an important role in improving gut barrier integrity, and regulating glucose and lipid metabolism as well as the immune system [26, 27]. Importantly, SFCAs inhibit LPS -induced inflammation through histone deacetylase and G-protein-coupled receptors [28, 29]. Besides, SFCAs process anti-inflammatory activities that regulate the expression of adhesion factors, pro-inflammatory cytokines, and chemokines [30, 31], such as tumor necrosis factor-α (TNF-α), IL-1β, IL-6, and IL-10 [32, 33]. Furthermore, SCFAs may be able to prevent myometrial contractions and rupture of membranes. The SCFAs butyrate and propionate suppressed inflammation-induced expression of the uterotonic prostaglandin PGF2alpha, myometrial cell contraction, and enzymes involved in remodeling myometrium and degradation of the fetal membranes [34].

Although many causes of PTB have been identified, accumulating evidence indicates that parturition is an inflammatory process [35]. PTB is firmly linked to inflammation regardless of infection [36]. Premature activation of the uterus by inflammation may lead to PTB, and IL-1 has been implicated in the cascade of events leading to PTB [37]. Multiple harmful factors stimulate the maternal innate immune response by activating Toll-like receptors, leading to the production of pro-inflammatory cytokines [38]. The cytokines subsequently activate gestational tissue, augment the contractility of the uterine, and drive cervical dilation and rupture of the fetal membrane [7, 34]. Animal experiments and human studies have shown that NLRP3 (NLR family pyrin domain-containing protein 3), caspase-1 (CASP-1), and IL-1 β were a high expression in amniotic fluid, amniotic membrane, and basement membrane detachment of premature mothers [39–41]. The IL-1β and TNF-α augment myometrial contractility by increasing calcium entry into myometrial smooth muscle cells [42]. In addition, as shown in mice, IL-1 administration induces PTB, inhibiting its receptor prevents labor [43]. An IL-1 receptor-associated kinase 1 (IRAK1) inhibitor significantly decreased PTB and increased live birth in WT mice [44]. The IL-1 receptor inhibitor, rytvela, may be of use in resolving inflammation associated with PTB and fetal injury [36, 45].

Inflammation is recognized as the major underlying cause of PTB, and SFCAs produced by *Lachnospiraceae* can inhibit inflammation through different signaling pathways. At the same time, the gut of mothers delivering prematurely shows a significant reduction of SFCAs-Producing microbiomes. The dysbiosis of the maternal gut microbiomes may affect fetal birth outcomes through microbiome-generated metabolites. Nickodem et al. [46] used plasma from 20 premature mothers and 30 healthy full-term mothers at the time of admission to labor and delivery to examine the maternal SFCAs to pregnancy outcome, founding that propionic acid in maternal plasma was negatively correlated with premature delivery. Although this is a pilot study, it provides an inspection to explore whether bacterial metabolites protect against inflammation pathways during pregnancy and reduce the incidence of PTB.

The present study has several limitations. First, the samples of preterm mothers were relatively small. When the sample sizes of two groups are significantly different, the efficacy of the t-test may be reduced. It may be more appropriate to use an alternative statistical method better equipped to handle uneven data, such as a non-parametric test like the Wilcoxon rank-sum test. Consequently, we implemented the Kruskal-Wallis test and the Wilcoxon rank-sum test for group comparisons. While these tests can partially mitigate the effects of sample size inequality, they cannot entirely eliminate the potential bias resulting from the imbalance. It is better to balance the sample sizes as much as possible. Secondly, we did not collect the dietary data. Although all the participants were from Chengdu (China), and generally show similar dietary habits, with a preference for spicy foods. It is known that dietary influences the gut microbiome. Therefore, it is important to collect and analyze dietary data in future studies.

So in the future, we plan to enroll a larger population to validate our findings. Secondly, we have observed that the mothers delivering preterm show a decrease of *Lachnospiraceae* which produces SFCAs. Therefore, we would like to explore whether this reduction of *Lachnospiraceae* corresponds to a decrease of SFCAs in the circulation of preterm mothers. At the same time, the dietary is an important factor, we will collect the dietary data of mothers to analyze the influence of dietary.

## Conclusion

In summary, applying longitudinal sampling and whole-genome shotgun metagenomic analysis on 80 gut microbiotas of 43 mothers, we demonstrate that the disrupted gastrointestinal bacteria in mothers delivering prematurely, particularly the lack of *Lachnospiraceae*, predisposed fetus to the risk of PTB. This highlights the need for large-scale animal models to understand the impact of the maternal gut microbiome on newborn outcomes.

## Electronic supplementary material

Below is the link to the electronic supplementary material.

Supplementary Material 1

## Data Availability

The datasets used and/or analysed during the current study are available from the corresponding author on reasonable request. The raw data have been submitted to China National GeneBank DataBase, CNGBdb (accession number, CNP0003746).

## List of abbreviations

PTB: Preterm birth
SFCAs: Short fatty chain acids
IL-1: Interleukin-1
LEfSe: Linear discriminate analysis effect size
IRAK1: IL-1 receptor-associated kinase 1
TNF- α: Tumor necrosis factor-α
CASP-1: caspase-1
