# Supplementary material for: Prematurely delivering mothers show reductions of lachnospiraceae in their gut microbiomes
Source: BMC Microbiol. 2023 Jun 15;23:169. doi: 10.1186/s12866-023-02892-z (PMC10268532; doi:10.1186/s12866-023-02892-z)
Supplement: Supplementary file 1 — Supplementary Material 1 [file 12866_2023_2892_MOESM1_ESM.docx]

**Supplementary Figure**


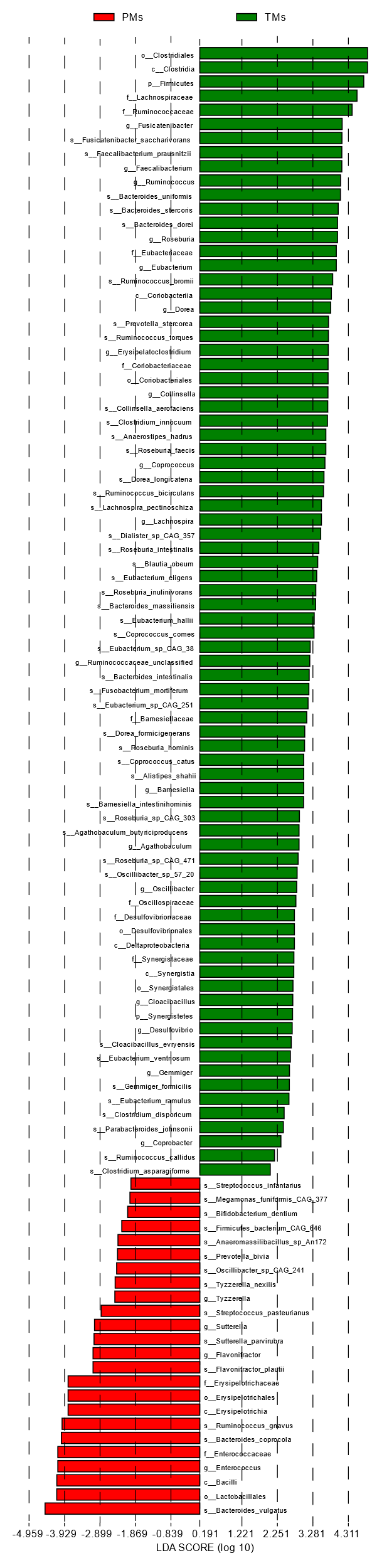


**Figure S1** Taxa identified by LEfSe as differentiating between PMs and TMs, the influencing degree of taxa was expressed by the length of bar in histogram, the criteria for feature selection is log LDA score >2
